# Munchausen Syndrome by Proxy Presenting as Pharyngeal Dysphagia and Recurrent Mouth Ulcers: A Case Report

**DOI:** 10.7759/cureus.49154

**Published:** 2023-11-20

**Authors:** Fadiah Alkhattabi, Sulaiman Alsalameh, Abdullah Alkhani, Raghad Alhuthil, Ahmad Hatem, Alaeddin Jebreel

**Affiliations:** 1 Department of Pediatrics and Child Health, King Faisal Specialist Hospital and Research Centre, Riyadh, SAU; 2 College of Medicine, Alfaisal University, Riyadh, SAU; 3 Department of Pediatrics, King Faisal Specialist Hospital and Research Centre, Riyadh, SAU; 4 Department of Otolaryngology, King Faisal Specialist Hospital and Research Centre, Riyadh, SAU

**Keywords:** bleeding, recurrent mouth ulcers, child abuse, pharyngeal dysphagia, munchausen syndrome by proxy

## Abstract

We report a Munchausen syndrome by proxy (MSBP) case, which presented as pharyngeal dysphagia and an acquired tracheoesophageal fistula (TEF).

A six-month-old Saudi male presented with fever, persistent oral ulcers, intermittent bleeding from the ulcers, failure to thrive (FTT), poor appetite, and possible genetic disease. He had a history of recurring admissions due to infections, including those affecting the chest, ear, and bowel. Additionally, he tested positive for vancomycin-resistant enterococcus. There was no history of surgical procedures or blood transfusions. Due to the patient’s nutritional status, a gastrostomy tube was inserted. The patient had recurrent bleeding from the tracheostomy tube during the hospital stay despite normal coagulation and platelet profile. Consequently, after diagnostic laryngoscopy, the otolaryngologist specialist pointed out that such retropharyngeal injuries are seen in patients with inflicted injuries, which is, in this case, caused by the mother, as she was the only one with the child during the recurrent bleeding episodes.

Thus, we describe a clinical instance of MSBP, especially imitating pharyngeal dysphagia, leading to a delayed diagnosis. We advise adding MSBP to the possible diagnoses upon encountering pharyngeal dysphagia and oral ulcers.

## Introduction

Pharyngeal dysphagia, also known as oropharyngeal dysphagia, is a swallowing disorder that affects the coordination and function of the muscles involved in swallowing in the oropharynx [1]. It is characterized by difficulty initiating or completing the swallowing process, which can lead to a range of complications, including malnutrition, aspiration pneumonia, and reduced quality of life [2]. Understanding the underlying causes, clinical manifestations, and diagnostic approaches is essential for effective management and improved patient outcomes [2,3].

Pharyngeal dysphagia can occur at any age and can be either acute or chronic [1]. The common causes of pharyngeal dysphagia include neurological conditions, such as stroke, Parkinson’s disease, and multiple sclerosis, as well as structural abnormalities, such as head and neck cancers, Zenker’s diverticulum, or postsurgical complications [2]. It can also be caused by muscular disorders, such as myasthenia gravis or muscular dystrophy; identifying the underlying cause is crucial as it guides appropriate management strategies [2,3]. Therefore, we report an unusual case that presented as pharyngeal dysphagia and acquired tracheoesophageal fistula (TEF).

## Case presentation

A six-month-old Saudi male presented with a fever for six days (tympanic temperature: 39°C), persistent oral ulcers (more than 10 days), intermittent bleeding from the ulcers, and poor appetite. He was referred for possible immunodeficiency or genetic disease. He is a product of full-term pregnancy and was born via spontaneous vaginal delivery, with no complications during pregnancy; his birth weight was about 3 kg, with no neonatal intensive care unit (NICU) admission. He had delayed umbilical cord separation for 25 days with infection and pus that improved with topical antibiotics, as reported by the mother and clinical notes. He was diagnosed with glucose-6-phosphate dehydrogenase (G6PD) deficiency at two months of age. He had a history of recurrent admission for infections (the chest, ear, and bowel), and he is positive for vancomycin-resistant enterococcus. There was no history of operation or blood transfusion.

The patient was doing fine and thriving until four months of age when he started to have severe bloody mucoid diarrhea and poor weight gain. He was treated with a course of antibiotics at a local hospital. He had four other upper respiratory tract and gastrointestinal infections that required two courses of oral and intravenous (IV) antibiotics, respectively. Accordingly, the patient developed reactive airway disease and was treated with low-dose inhaled corticosteroids and as-needed Ventolin. The patient was also advised to stay away from certain foods and medications as he developed jaundice and splenomegaly after certain medications were taken at a local hospital.

**Figure 1:**
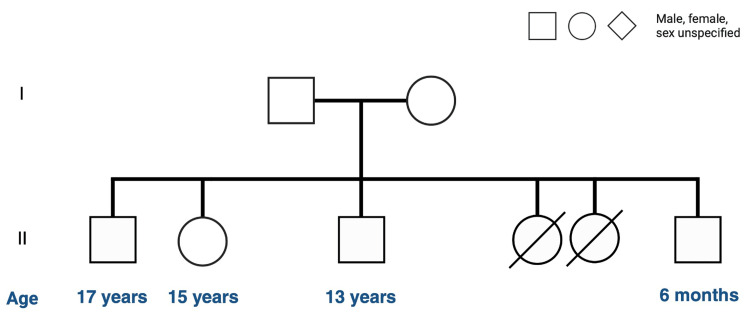
The family pedigree of the patient. There is a history of two female siblings who died when they were four and 3.5 years old with infections and bleeding (no clear diagnosis as reported by the mother and no autopsy was done from the referring hospital), and the patient has three healthy siblings, one female and two males. There is a history of bronchial asthma in the mother and older brother; there were no other familial diseases.

Regarding the family history, there is no reported consanguinity. However, it is noteworthy to mention that the father has multiple wives and over 20 children. The mother also mentioned that two female siblings, aged four and 3.5 years, passed away due to infections of unclear origin (see Figure 1). The mother, who is a 42-year-old housewife with a high school degree, comes from a poor socioeconomic status. She has hypertension and bronchial asthma, and the father has diabetes. The patient is meeting his developmental milestones in all areas. Thus, he was referred to our hospital for further investigations.

**Figure 2:**
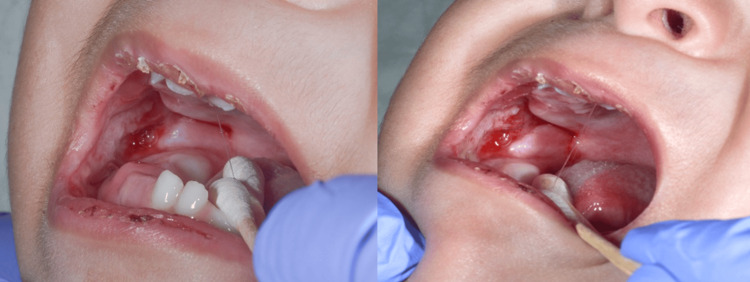
Right buccal mucosal bleeding with no signs of pus or infectious process.

**Figure 3:**
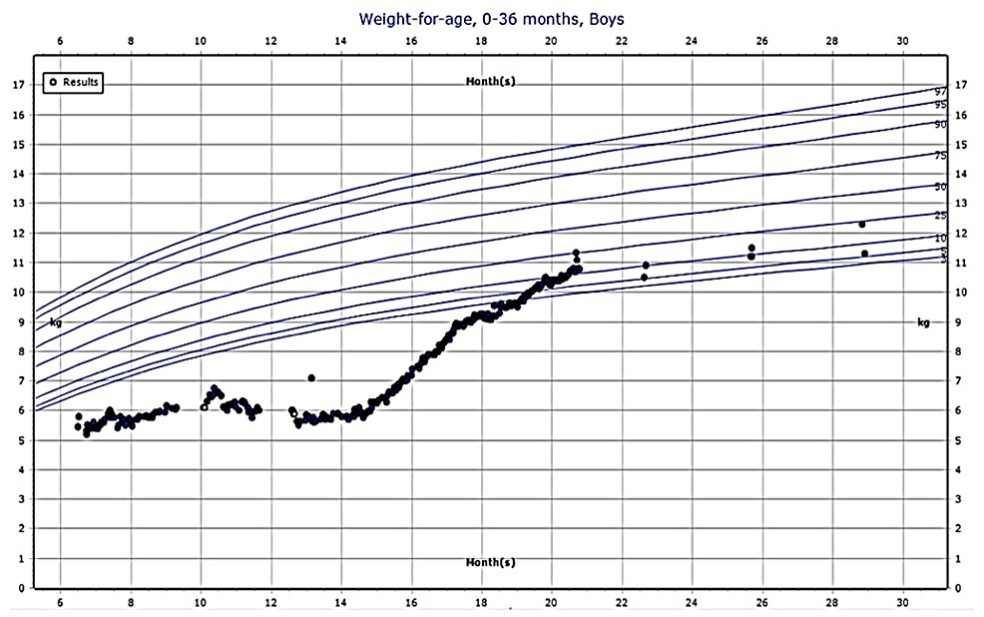
Growth chart showing how initially the patient was below the third percentile for weight-for-age. At 15 months of age, the child starts gaining weight after gastrostomy tube insertion. Later at 21 months of age, it was removed, and the child maintained acceptable weight as the mother was separated.

Upon presentation at the emergency department, he was initially admitted with retropharyngeal inflammation and recurrent mouth ulceration (see Figure 2). Moreover, according to the growth chart, he had more pronounced poor weight gain, which met the criteria for failure to thrive (FTT) (weight is 5.45 kg below the third percentile) (see Figure 3). The initial workup that was done excluded primary immunodeficiency and metabolic diseases. Therefore, the patient was discharged after around nine months of hospital stay (from November to July). However, a few days after discharge, the patient was readmitted for the second time to our emergency department; he was 15 months of age. He was found to be febrile with a temperature of 39°C, with actively bleeding mouth ulcers and a wet cough. Multiple services, including otolaryngology, infectious diseases, immunology, and rheumatology, extensively investigated him. Multiple meetings were held, and the possibility of hereditary hemorrhagic telangiectasia (HHT) and auto-inflammatory diseases was ruled out due to negative results.

Unluckily, after one month, the mouth ulcers were severely infected, which caused acute retropharyngeal edema requiring broader-spectrum antibiotics and tracheostomy tube insertion due to upper airway obstruction, which required intubation for four days. He stayed in the pediatric intensive care for around 10 days. The patient also had cytomegalovirus (CMV) (quantification: 156 copies/mL) that responded well to the valganciclovir course.

**Figure 4:**
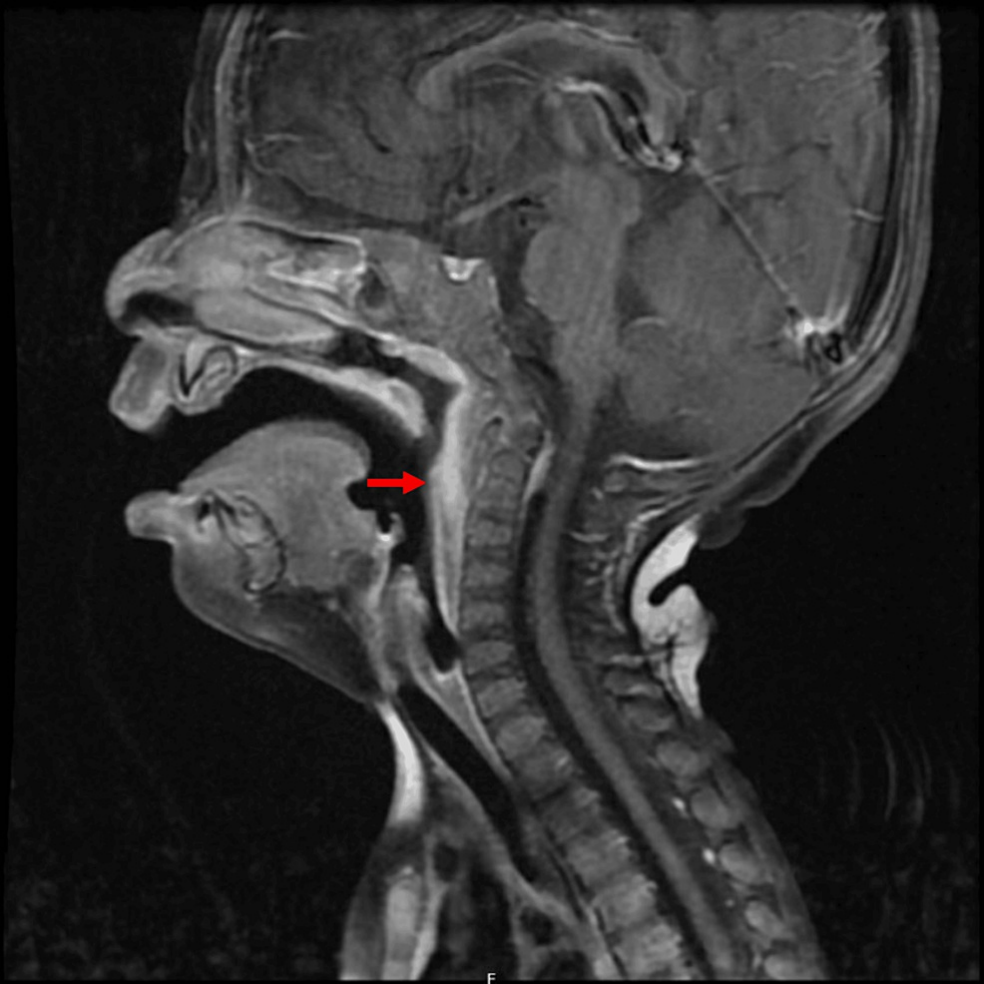
Magnetic resonance imaging (MRI) T1 with contrast coronal view, showing diffuse thickening, edema, and prominent enhancement of the retropharyngeal and prevertebral soft tissues with associated edema.

The head and neck magnetic resonance imaging (MRI) with contrast revealed diffuse thickening, edema, and prominent enhancement of the retropharyngeal and prevertebral soft tissues, with associated edema; the abnormality is relatively symmetrical without discrete mass lesion (see Figure 4).

Due to the patient’s nutritional status, a gastrostomy tube was inserted. The patient had recurrent bleeding from the tracheostomy tube during the hospital stay despite the normal coagulation and platelet profile (the international normalized ratio {INR} is 1.1, reference range: 0.8-1.1; platelet: 432 × 109/L, reference range for age: 155-435).

The following multidisciplinary meeting involving the otolaryngologist specialist after diagnostic laryngoscopy pointed out that such retropharyngeal injuries are seen in patients with non-accidental injuries. This raised the suspicion of child abuse by the mother, as the recurrent bleeding episodes happened when the mother accompanied the child. Adding to that, whenever discharge planning was discussed with the patient’s mother, the incidence of hematemesis increased.

The mother was confronted, but she did not disclose or admit her actions. Therefore, the pediatrician explained to the patient’s father the possibility of Munchausen syndrome by proxy (MSBP), and the father agreed to move forward with the necessary steps. The hospital’s social services and the Ministry of Social Affairs were involved in the parent-child separation process. The hospital arranged a babysitter for the patient, and the patient’s room was changed daily to prevent the mother from visiting. After three weeks, no more bleeding was noticed from the tracheostomy tube. Four months later, all oral ulcers were resolved; the tracheostomy and gastrostomy tubes were decannulated, and the patient’s CMV quantification was no longer detectable.

At four years of age, the patient came for follow-up to the child protective clinic accompanied by the father, sister, and aunt. Clinically, the patient was medically free and meeting his developmental milestones and growth parameters (height standard deviation score {SDS} score, 0.02; weight SDS, 0.21), which are both within the normal range (5th-95th percentiles). Socially, the patient was living with his aunt and sister. The mother was evaluated for psychiatric disorders in another hospital (mental institute) and was admitted for one week, as reported by the husband, while maintaining no interaction with her child. However, the mother was not compliant with her psychiatric help. The father and sister were counseled on the importance of maintaining the child’s safety, and the patient was discharged as the treatment plan was completed.

## Discussion

MSBP is a term used to describe abuse where the abuser is diagnosed with a factitious disorder imposed on another (FDIA) due to a caregiver-fabricated illness [4,5]. A systematic review of 796 cases showed that nearly all abusers were female (97.6%), most commonly the victim’s mother (95.6%) [5]. Isolating the child from the parent has proven to be an effective diagnostic technique [4]. The key to diagnosis was symptom resolution after a parental absence, which was a recurrent finding, particularly in fabrication cases in the medical literature [5,6].

Furthermore, according to the American Professional Society Abuse Children (APSAC), the separation of the child from the suspected abuser for a period of time is a diagnostic criterion for MSBP, as it often leads to the resolution of symptoms [7].

The current case is an unusual presentation of MSBP. According to Rees et al.’s systematic review of otolaryngologic manifestations of child abuse, pharyngeal perforations presenting with dysphagia and surgical emphysema were reported in 20 out of 122 cases of maltreated children [8].

The current case bears similarities to a previous case documented in our hospital back in 1993, as reported by Al-Jumaah et al. In this prior case, a 15-month-old Saudi female had been in good health until the age of 13 months when she suddenly developed symptoms such as fever, cough, and the rapid onset of ulcerative lesions primarily affecting her oral cavity and lips [9]. Clinical examination revealed a distressed child who was drooling, with fresh ulcers on the oral mucosa and corners of the mouth, extending into the infra-auricular area. These lesions were indicative of an acute chemical injury and strongly suggested deliberate harm. Upon confrontation, the mother admitted her actions, and she was subsequently referred for psychiatric support [9].

A few other cases regarding MSBP in Saudi Arabia were reported, including a report by Al-Mugeiren and Ganelin about a 17-month-old Saudi male who presented with recurrent episodes of hematemesis, bleeding per rectum, and hematuria [10]. In addition, Alkhattabi et al. reported a case of factitious hypoglycemia [11]. Overall, these case reports collectively underscore the presence of MSBP in Saudi Arabia and provide valuable clinical insights. While each study offers a unique perspective, they all highlight the importance of early recognition, multidisciplinary collaboration, and appropriate legal measures in managing cases of MSBP.

To solve instances with unusual combinations of symptoms and a poor response to anti-inflammatory medications, one must have a high suspicion index for MSBP. In our case, the patient had a fever, recurrent mouth ulcers, FTT, and recurrent chest, ear, and bowel infections at admission, and due to his nutritional status, a gastrostomy tube was inserted.

Fortunately, the tracheostomy tube did help save his life during the acute bleeding episodes. Moreover, after diagnostic laryngoscopy, the otolaryngologist specialist pointed out that such retropharyngeal injuries are seen in patients with non-accidental injuries.

Therefore, it is essential to be aware of presentation patterns and parental behavior to diagnose MSBP early and prevent unnecessary diagnostic and surgical treatments [6]. Pharyngeal or external ear injuries in young children without a detailed history of witnessing harm should elicit a referral to the child protection service for a comprehensive evaluation, and consultation with a child safety specialist is advised because they have the potential to identify these children in their practice, recognizing possible child maltreatment and initiating appropriate safeguarding measures [6].

## Conclusions

The timely recognition of MSBP is important for early intervention and preventing further complications. The accurate diagnosis of MSBP requires a comprehensive evaluation that may involve multidisciplinary healthcare professionals, including pediatricians, otolaryngologists, radiologists, and social workers. Clinical assessment, taking comprehensive patient history through physical examination, laboratory investigations, and radiology images are particularly important when having an unusual combination of symptoms. These procedures help identify the underlying cause of these symptoms and guide the management plan. Thus, it is essential to include MSBP as a differential diagnosis in children with recurrent unexplained bleeding or infected mouth ulcers.
